# Youth health behaviors and their reverse intergenerational impact

**DOI:** 10.3389/fpsyg.2025.1631102

**Published:** 2025-09-25

**Authors:** Shaoang Xia, Ke Zhang, Chuwen Yan, Yayu Zhou, Chunqu Xiao

**Affiliations:** ^1^School of Law and Institute of Chinese Legal Modernization Studies, Nanjing Normal University, Nanjing, China; ^2^School of Management and Engineering, Nanjing University, Nanjing, China; ^3^Business School, Jinling Institute of Technology, Nanjing, China; ^4^School of Business, Shaoxing University, Shaoxing, China

**Keywords:** cultural feedback, health behaviors, misinformation, reverse intergenerational influence, social media

## Abstract

**Introduction:**

In the context of rapid population aging and digital transformation in China, youths are increasingly influencing older family members' health perceptions and behaviors through “cultural feedback” mechanisms. This study examines the factors shaping youth health behaviors and their reverse intergenerational effects on elders.

**Methods:**

A cross-sectional survey of 2,187 participants aged 18–35 examined factors influencing youth health behaviors and their reverse intergenerational influence on older family members, using regression analyses with mediation models.

**Results:**

Findings indicate emotional regulation capacity emerged as the strongest predictor of health behaviors, followed by surrounding individuals‘ health status, gender, and self-reported health condition. Socioeconomic status, occupation, and age demonstrated limited direct effects. Young adults' health behaviors significantly predicted reverse intergenerational influence, with health behavior tendency mediating relationships between individual characteristics and intergenerational influence.

**Discussion:**

Young adults serve as pivotal agents in health-related “cultural feedback”. Their health behavior tendencies subsequently influence older generations' health perceptions and practices. Interventions targeting emotional regulation skills among young adults may effectively amplify intergenerational health promotion while addressing aging-related public health challenges.

## 1 Introduction

### 1.1 Background

According to the Statistical Report on China's Internet Development Status, as of December 2024, internet penetration among those aged 60 and above reached 52.5%. However, while enjoying digital convenience, these populations confront challenges with online misinformation and rumors (Wei and Sang, 2019). Self-media and short-video apps expose older users to mixed information that can confuse health concepts when their digital literacy is limited. The echo chamber effect of online health information may cause information fatigue among older adults, leading to avoidance behaviors that further weaken their ability to make informed health decisions (Zhong and Gu, 2024).

In response to these challenges, young people in many families are becoming “rumor busters” and “advocates” of health behaviors. This phenomenon manifests as young adults use internet literacy and scientific reasoning to identify health misinformation while serving as family-level health promoters and corrective agents. Zhou (1988) terms this reverse intergenerational influence “cultural feedback”—the process where older generations absorb cultural elements from younger generations during rapid social change. Young people leverage digital literacy and scientific knowledge to identify health misinformation and locate credible sources (Zhou, 2011). Recent research indicates that digital technologies enhance learning engagement—a finding that parallels how youth utilize interactive platforms to convey health information to older generations (Cheng et al., 2025).

This dynamic reveals a fundamental generational divide in information processing: older adults demonstrate greater vulnerability to misinformation, while younger individuals actively engage in debunking efforts. Consequently, youth have emerged as household-level health advocates and misinformation mediators, fostering intergenerational health dialogue across age groups from Generation Z to the elderly.

The mechanisms behind youth health behaviors and their reverse intergenerational effects remain unclear. Individual characteristics such as emotional regulation capacity, socioeconomic factors, and social environmental contexts may influence health behavior formation, which subsequently affects older family members through modeling, information transmission, and resource provision pathways. Understanding these mechanisms could address national health literacy challenges through intrafamily interventions, leveraging youth as change agents for health promotion.

### 1.2 Theoretical framework and hypotheses

#### 1.2.1 Health behaviors and their influences

The World Health Organization (n.d.) recognizes that health behaviors are complex, influenced by knowledge, motivation, social norms, cultural contexts, and resources. This study primarily draws on social cognitive theory and social ecological theory to construct its theoretical framework, aiming to explore the mechanisms influencing youth health behaviors across three dimensions: individual, socioeconomic, and social environmental.

Social cognitive theory posits emotional regulation as central to behavior control, affecting choices under stress (Gross, 2002). Mechanistically, it influences self-regulation resources and coping strategies, enabling effective resource management (Muraven et al., 1998). Strong emotional regulation correlates with healthier lifestyles, such as keeping physical exercise, avoiding risky behaviors, and following medical advice (Denollet et al., 2008). Conversely, poor regulation links to unhealthy behaviors in youth (Gianini et al., 2013). Furthermore, in the context of major public health crises, poor emotional responses not only impair immune functions but also lead to more destructive interpersonal interactions (Liang, 2003), setting off a vicious cycle that amplifies health risks. In summary, emotion regulation ability is a potent predictor of health behaviors in youth, prompting the hypothesis that:

Gender and age are key demographic variables. Gender socialization leads to systematic differences, with females showing more health-conscious behaviors and males potentially rejecting them to affirm masculinity (Courtenay, 2000). Females engage more in check-ups, healthy diets, and exercise; whereas males—due to societal expectations—may delay treatment or overlook preventive measures (Galdas et al., 2005). Although young males' health awareness is rising, gender differences in health behaviors and outcomes remain pronounced (Su et al., 2022). Thus:

Age reflects developmental stages, with growth enhancing cognition, risk perception, and self-control (Moffitt et al., 2011). Longitudinal studies show age-related declines in problem behaviors and increase in protective ones (Jessor et al., 1998). Per social cognitive theory, age boosts perceived behavioral control and self-efficacy (Bandura, 1977). Consistent with this reasoning, empirical evidence shows that self-efficacy is positively associated with sustained health behaviors and that this association is more pronounced among older youth (Schwarzer and Renner, 2000). Thus:

Socioeconomic status (SES) affects lifestyles and health outcomes. Populations of lower SES face higher health risks, attributable not only to material deprivation but also to poor health behaviors (Marmot, 2005). Higher SES typically correlates with healthier diets, exercise, and lower risk behaviors (Adler and Newman, 2002). Social cognitive theory explains this via enhanced knowledge, resources, and self-efficacy (Pampel et al., 2010). Furthermore, research suggests that this health disparity caused by SES is cross-cultural (Kunst et al., 2005). Thus:

The relationship between occupational status and health behavior presents a relatively complex picture. Working youth face pressures that challenge health maintenance (Burke et al., 2004) yet transitions from student to worker often improve diets and activity (Winpenny et al., 2020). This may stem from “role strain”. The transition from student to worker often comes with enhancement of self-management (Blokker et al., 2023). Social responsibilities and professional expectations increase motivation for health maintenance. Per conservation of resources theory, health is vital for job performance (Griep et al., 2016). Lastly, workplace health promotion programs and occupational health regulations provide additional incentives for healthy behaviors (Rongen et al., 2013). Thus:

The health status of people surrounding provides social comparison cues, thereby influencing individuals‘ health behaviors via observational learning. Two competing theories account for this influence. Christakis and Fowler (2007) suggests that obesity is “contagious” (or spreading) within social networks by altering personal acceptance of norms or directly influencing health behaviors. This mechanism can propagate both negative and positive health behaviors (Smith and Christakis, 2008). Conversely, the health promotion model suggests that observing others' negative health outcomes heightens perceived susceptibility and severity of health threats, serving as key motivation for health promoting behaviors (Zambrano-Bermeo et al., 2023).

These findings underscore an enduring tension within social cognitive theory: identical social cues may elicit opposite behavioral responses depending on individual cognitive processing patterns (Wills, 1981). Given that this study's measurement of “surrounding people's health” primarily captures individuals' exposure to health risks, the risk perception mechanism likely predominates. Therefore:

Intergenerational influence was viewed as a process in which older generations pass on values, knowledge, and social practices to younger generations. Contrary to the conventional pattern, China, especially after the reform and opening-up, has witnessed a marked bidirectional dynamic. Zhou (2000) terms this reverse intergenerational influence “cultural feedback,” defined as “the process where older generations absorb extensively cultural elements from younger generations, during rapid social change”. In this context, the younger generation, with its greater familiarity with new technologies, plays a significant role in influencing, and sometimes reshaping, the behaviors of their parents.

Reverse intergenerational influence represents a departure from traditional models of family socialization, where knowledge and behavioral patterns flow from younger to older family members rather than following conventional parent-to-child transmission pathways. Contrary to the conventional pattern, China, especially after the reform and opening-up, has witnessed a marked bidirectional dynamic. Zhou (2000) terms this reverse intergenerational influence “cultural feedback”, defined as “the process where older generations absorb extensively cultural elements from younger generations, during rapid social change”. In this context, the younger generation, with its greater familiarity with new technologies, plays a significant role in influencing, and sometimes reshaping, the behaviors of their parents.

Drawing from Bandura's (1977) social learning theory, intergenerational influence typically operates through modeling, where parents serve as primary behavioral exemplars for children. However, “cultural feedback” inverts this dynamic, positioning young adults as health behavior models and information intermediaries for their parents. Unlike traditional intergenerational transmission patterns or simple shared family health behaviors that occur simultaneously without clear direction (Wickrama et al., 1999), reverse influence involves deliberate, goal-directed attempts by younger members to modify parental behaviors through their superior access to digital health resources, updated health knowledge, and technological competency. Reciprocal communication patterns in families, as described by Gong et al. (2023), involve bidirectional exchanges where all family members mutually influence each other's health decisions. In contrast, “cultural feedback” specifically emphasizes the younger generation's role as primary change agents, leveraging their technological competency and updated health knowledge to guide parental behavior modification.

In summary, this study examined the role of socioeconomic status, emotional management skills, gender, age, occupation, health level of those around them, and perceived level of self-health on health behaviors among youth groups. Accordingly, the following research hypotheses were formulated:

**Hypothesis 1**: Youth socioeconomic status positively influences youth health behaviors.

**Hypothesis 2**: Youth emotional control positively influences youth health behaviors.

**Hypothesis 3**: Female youth are more conscious of health behaviors than male youth.

**Hypothesis 4:** Youth age positively influences health behaviors.

**Hypothesis 5:** The working population is more focused on health behaviors than the student population.

**Hypothesis 6:** The health and self-health perceptions of those around them positively influence youth health behaviors.

##### 1.2.1.1 Mechanisms of reverse intergenerational influence

This paper argues that the reverse intergenerational influence is more pronounced in the transmission of health concepts at the family level. Family health socialization traditionally conceptualized parents as primary health socializing agents for children (Morris et al., 2007). Reverse intergenerational influence represents a role reversal within this framework, where adult children assume the socializing role for their parents, particularly in contexts involving new health technologies or updated health guidelines. The “cultural feedback” hinges on the integration of emerging technologies. Because young people adopt new technologies more readily than their elders, each new generation of technology may further widen the digital divide between them.

The digital divide manifests not merely in access and usage levels, but more significantly in disparities in digital skills and usage patterns (Van Deursen and Van Dijk, 2009). In the context of health promotion, this divide operates on multiple levels. First, differences exist in health information acquisition capabilities, with younger generation demonstrating proficiency in obtaining relevant information (Norman and Skinner, 2006); Second, disparities emerge in information evaluation abilities, where younger individuals show greater competence in identifying misinformation (Swire-Thompson and Lazer, 2020). This intergenerational disparity in digital literacy creates a knowledge power inversion.

The digitization of health services further amplifies this dynamic. Younger generation may assume the role of “health gatekeepers” for their parents, filtering health information and monitoring health behaviors, thereby establishing novel patterns of reverse intergenerational influence. This technological dependency creates structural opportunities for young adults to influence parental health behavior through information provision and technological mediation. Moreover, the proliferation of unverified health information in digital media environments makes parents particularly vulnerable to misinformation, creating an urgent need for younger family members to serve as information filters and “rumor busters” (Zhao, 2022). Young adults with strong health behavior tendencies are better positioned to fulfill this protective role effectively.

##### 1.2.1.2 Youth health behavior tendency as mediating mechanism

Health behavior tendency represents an individual's propensity to adopt and sustain health-promoting behaviors. Within cultural feedback dynamics, youth health behavior tendency affects not only their own health but, more critically, their parents. Social cognitive theory's reciprocal determinism posits dynamic interactions between individual characteristics, behavioral performance, and environmental factors (Bandura, 1989). Within the reverse intergenerational influence framework, youth health behavior tendency serves as a critical mediating variable linking individual characteristics and environmental factors to family health outcomes. The mediating role may operate through four pathways:

In reverse intergenerational influence, individual's characteristics (socioeconomic status, emotional regulation, gender, age, occupation) and environmental factors (social health contexts, health self-perception) shape health behavior tendencies, which subsequently influence parents' health behaviors through several mechanisms:

First is the information capability pathway. Young adults with strong health behavior tendencies actively seek, evaluate, and synthesize health information, serving as family health information gatekeepers while filtering potentially harmful misinformation. Second is behavioral modeling. According to social cognitive theory, role model behavior has important influence on observers (Nabavi, 2012). Youth with strong health behavior tendencies demonstrate observable health practices for parents. When parents witness positive outcomes from their children's health behaviors, they develop stronger motivation for behavioral change. Third, health behavior tendency enhances communication effectiveness within families. Young adults with genuine commitment to health behaviors communicate more authentically about health topics, increasing message credibility and persuasive impact. Additionally, the resource support pathway cannot be overlooked. Youth with high health behavior tendencies are more willing to invest time, energy, and economic resources to support family health, and are more likely to purchase health management equipment for parents, accompany parents in fitness activities, or bear the costs of health checkups. This instrumental support may directly reduce barriers for parents to adopt health behaviors.

Multiple factors contribute to youth health behavior tendency development, which subsequently influences reverse intergenerational effects. Higher socioeconomic status provides access to superior health information sources and resources, enhancing both health behavior tendency and capacity to support parental behavior change (Adler and Newman, 2002). Effective emotional regulation facilitates health behavior maintenance while improving family health communication effectiveness (Gross, 2015). Demographic factors including gender, age, and occupational status contribute through social role expectations and opportunity structures (House et al., 1988). Based on this theoretical framework, we propose:

**Hypothesis 7:** Youth health behavior tendency mediates the relationship between reverse intergenerational influence and the following variables: (a) emotional regulation ability; (b) socioeconomic status; (c) gender; (d) age; (e) occupational status; (f) surrounding people's health levels.

## 2 Materials and methods

### 2.1 Participants

This study uses a questionnaire survey method conducted via the professional data collection platform Questionnaire Star (Sojump). A total of 9,844 visits were made to the questionnaire, with 3,775 responses collected, resulting in a completion rate of 37.81%. Of these responses, 3,722 were valid, and 53 invalid questionnaires were eliminated. The sample covers all 30 provinces in the country. Data collection took place from February 14 to 22, 2020, spanning 9 days. The study focused on youth born between 1985 and 2003. As a result, the study limited the sample to individuals aged 18 to 35, yielding a final valid sample size of 2,187.

### 2.2 Measures

#### 2.2.1 Health behaviors

This study referenced the Integrated Behavioral Model literature to establish indicators for examining specific health behaviors. According to its theoretical framework, health behavior measurement indicators should be specific and clearly defined across four dimensions: action, target, context, and time (Montano and Kasprzyk, 2015). This study combined China's public health practices and residents‘ daily life realities, selecting four types of common health behaviors as measurement indicators: personal hygiene behaviors (maintaining basic hygiene habits such as regular handwashing and teeth brushing), physical activity behaviors (proactive health promotion activities such as outdoor exercise), health protection behaviors (such as wearing masks), and healthy eating behaviors (nutritional management behaviors such as calorie control). Additionally, research points out that reducing car use and increasing walking and cycling as “active commuting” has multiple benefits at physiological, psychological, and social levels as a health behavior intervention strategy (De Nazelle et al., 2011). Therefore, this study also included reducing “car dependency” as a health behavior indicator. All five specific items used a seven-point Likert scale, with options assigned scores from 1 to 7 (1 = very non-compliant, 7 = very compliant), using the mean score of these five items as an indicator of respondents' health behaviors. Cronbach's alpha coefficient for these five items was 0.866, indicating good reliability.

The questionnaire used three items to measure youth influence on elders' health concepts: “I can easily persuade elders to adopt health behaviors,” “With my persuasion, elders realize the importance of health,” and “Elders trust my words more than information from other sources.” A seven-point scale was still used, with respondents rating whether these descriptions matched their actual situations, with 1 representing completely non-compliant and 7 representing completely compliant. The Cronbach's alpha coefficient for these three items was 0.834, indicating good reliability, with the mean of these three items representing the magnitude of reverse intergenerational influence.

#### 2.2.2 Socio-economic status

This study measured both participants' subjective and objective socioeconomic status. Subjective socioeconomic status was measured by asking participants about their satisfaction with their economic status. Participants needed to choose from options on a seven-point Likert scale, with options assigned scores from 1 to 7 (1 = completely dissatisfied, 7 = completely satisfied). Higher scores indicated higher satisfaction. Objective socioeconomic status used annual household income as an indicator.

The measurement of subjective socioeconomic status through economic satisfaction alone may not comprehensively capture participants' socioeconomic circumstances. However, given the nationwide scope of this study, subjective SES measurement offered practical advantages including simplified administration and reduced participant burden compared to detailed objective SES assessments (Zhao et al., 2023). Nevertheless, subjective SES has demonstrated independent predictive validity for health outcomes beyond objective measures, with meta-analytic evidence showing unique associations with physical health above and beyond traditional SES indicators (Cundiff et al., 2017).

#### 2.2.3 Emotional management skills

Emotional management skills were adapted from three questions by Warren and Pezzuti (Warren et al., 2018): “I can control my emotions”, “I can keep my emotions from showing”, and “I can hide my emotions”. Respondents were asked to rate the extent to which these descriptions corresponded to their actual reality, using a scale from 1 to 7, with higher scores indicating greater conformity. The Cronbach's alpha coefficient for the three questions was 0.802, which provides good reliability. The mean value of the three questions was used as an indicator of emotion management ability.

#### 2.2.4 Other variables

Gender, age, occupation, health levels of surrounding people, and self-perceived health levels all have the potential to influence youth health behaviors. Gender and age were self-reported by respondents. For gender, males were assigned a value of 0 and females a value of 1. Occupation was a dichotomous variable that categorized respondents into student and non-student groups, assigning a value of 0 for student groups and 1 for non-student groups. The health level of surrounding people was measured on a continuous scale, ranging from “no sub-healthy groups around” to “I am a sub-healthy group,” divided into six levels reflecting the health status of individuals surrounding the respondents. Self-reported health level was assessed on a seven-point scale, with respondents rating their physical health, where higher scores indicated better health.

### 2.3 Measurement validation

To assess construct validity, we examined the psychometric properties of our key measures through reliability analysis and item-scale correlations. As shown in Table 1, all constructs demonstrated good internal consistency with Cronbach's alpha coefficients exceeding 0.80 (Health Behaviors α = 0.866, Reverse Intergenerational Influence α = 0.834, Emotional Regulation α = 0.802). Item-scale correlations ranged from 0.71 to 0.91, indicating that individual items correlated appropriately with their respective composite scales.

**Table 1:** Measurement items.

**Construct**	**Item**	**Item-scale correlation**	**Mean**	**SD**
**Health behaviors (**α = **0.866)**			6.47	0.88
	I wore a mask when going out	0.78^**^	6.65	0.99
	I try not to use public transportation	0.81^**^	6.51	1.11
	I try not to go out unnecessarily	0.85^**^	6.49	1.06
	I try not to contact people when going out	0.85^**^	6.48	1.06
	I wash my hands frequently	0.75^**^	6.24	1.22
**Reverse intergenerational influence (**α = **0.834)**			5.76	1.20
	I can easily persuade elders to adopt healthy behaviors	0.88^**^	5.85	1.36
	With my persuasion, elders realize the importance of health	0.87^**^	5.98	1.24
	Elders trust my words more than information from other sources	0.86^**^	5.45	1.53
**Emotional regulation (**α = **0.802)**			5.56^**^	1.25
	I can control my emotions	0.71^**^	5.97	1.33
	I can hide my emotions	0.91^**^	5.39	1.54
	I can keep my emotions from showing	0.90^**^	5.32	1.55

Items measured on 7-point Likert scales. *N* = 2,187. Item-scale correlations represent Pearson correlations between individual items and their respective composite scales.

^**^p < 0.01.

As previously mentioned, 7-point Likert scales measured health behaviors, reverse intergenerational influence, and emotional regulation. Compared with 5-point alternatives, 7-point scales reduce respondent interpolation and thus yield more accurate measurement (Finstad, 2010). This granularity was particularly important for detecting subtle differences in health behavior tendencies among Chinese youth. East-Asian respondents—including Chinese samples—tend to avoid extreme endpoints; an expanded midpoint range therefore retains meaningful variance that would otherwise be compressed (Chen et al., 1995). To address potential acquiescence bias, questionnaire items were carefully worded to be neutral and avoid leading phrasing, and response consistency was verified through high item-scale correlations and Cronbach's alpha values (see Table 1), indicating minimal bias in responses.

### 2.4 Data analysis

All statistical analyses were conducted using multiple regression with mediation modeling. For the mediation analysis, we employed bootstrap resampling procedures with 5,000 replications to generate bias-corrected confidence intervals for indirect effects, following recommended best practices for mediation analysis (Preacher and Hayes, 2008).

The mediation model tested the indirect effects of individual characteristics (emotional regulation ability, socioeconomic status, gender, age, occupation) and environmental factors (surrounding people's health status, self-perceived health) on reverse intergenerational influence through youth health behavior tendency. Bootstrap confidence intervals that do not contain zero indicate statistically significant mediation effects.

Standardized beta coefficients (β), *p*-values, and 95% confidence intervals (CIs) were reported for all predictors. Assumptions of normality, homoscedasticity, and multicollinearity were examined and met. All tests were two-tailed, and statistical significance was determined at the *p* < 0.05 level. In all tables, ^*^p < 0.10 (marginal significance), ^**^p < 0.05 (statistically significant), and ^***^p < 0.01 (highly significant).

## 3 Results

### 3.1 Health behaviors

This paper first analyzes the factors influencing youth health behaviors. Since this variable is continuous, hierarchical regression analysis is conducted, with health behavior as the dependent variable, subjective socio-economic status and emotional management ability as independent variables, and family income level, gender, age, occupation, health level of those around them, and perceived self-health level as control variables. Since there are some differences in social experience and perceptions between the student group and the working group, in the analysis of the full sample, we included occupation as a control variable in the full sample analysis and performed regressions for the student and non-student groups separately. Table 2 shows the regression results for the full sample, while Tables 3, 4 show the regression results for the student and non-student groups, respectively.

**Table 2:** The regression results for the full sample.

**Variables**	**Model 1**		**Model 2**		**Model 3**	
	**B**	**SE**	**B**	**SE**	**B**	**SE**
Emotional management skills	0.207^**^	0.015			0.208^**^	0.014
Subjective socio-economic status	0.019	0.011			00.016	00.012
Household income level	−0.026	0.016			−00.013	0.015
distinguishing between the sexes			0.221^**^	0.037	0.260^**^	0.035
(a person's) age			0.009	0.005	0.009	0.005
careers			−0.011	0.051	−0.062	0.050
Level of health of the surrounding population			0.076^**^	0.021	0.079^**^	0.021
Self-health level			−0.223 ^**^	0.032	−0.167 ^**^	0.031

Model 1 includes main predictors; Model 2 adds demographic and social variables; Model 3 presents the full model. Hereafter, standardized coefficients (B) and standard errors are reported in all tables unless otherwise specified.

^**^p < 0.01.

^*^p < 0.05.

**Table 3:** The regression results for the student population.

**Variables**	**Model 1**		**Model 2**		**Model 3**	
	**B**	**SE**	**B**	**SE**	**B**	**SE**
Emotional management skills	00.193^**^	00.026			00.211^**^	0.026
Subjective socio-economic status	0.076^**^	0.022			0.069^**^	0.022
Household income level	0.010	0.027			−0.006	0.027
Distinguishing between the sexes			0.276^**^	0.066	0.336^**^	0.064
(a person's) age			−0.010	0.014	0.001	0.013
Level of health of the surrounding population			−0.092	0.055	−0.109^*^	0.052
Level of self-health			−0.196 ^**^	0.064	−0.123^*^	0.062

The analysis excludes occupation variable as all participants are students.

^**^p < 0.01.

^*^p < 0.05.

**Table 4:** The regression results for the non-student groups.

**Variables**	**Model 1**		**Model 2**		**Model 3**	
	**B**	**SE**	**B**	**SE**	**B**	**SE**
Emotional management skills	0.217^**^	0.018			0.211^**^	0.017
Subjective socio-economic status	−0.004	0.013			−0.009	0.013
Household income level	−0.049^**^	0.019			−0.030	0.018
Distinguishing between the sexes			0.182^**^	0.044	0.192^**^	0.042
(a person's) age			0.013^*^	0.006	0.013^*^	0.005
Level of health of the surrounding population			0.113^**^	0.023	0.113^**^	0.022
Level of self-health			−0.231 ^**^	0.036	−0.186 ^**^	0.035

^**^p < 0.01.

^*^p < 0.05.

The estimation results show that emotional management ability plays a significant role in the health behaviors of youth, indicating that the stronger the emotional management ability, the more likely youth are to adopt healthy behaviors. Moreover, the standardized coefficient of emotion management ability is the highest in the regression model, and this result applies to both the student group and the working group, which indicates that emotion management ability has a greater effect on the health behaviors of the whole youth group. Occupation itself had a non-significant effect on health behaviors, indicating that youth in the student group and the work group equally valued health behaviors. In the full sample, the roles of subjective socioeconomic status and family income level were both insignificant. However, in the student group, subjective socioeconomic status positively predicted health behaviors, suggesting that students who perceived their current level of socioeconomic status to be higher were more likely to adopt healthy behaviors. Whereas, in the working group, the effect of subjective economic satisfaction was not significant, the level of actual family income had a significant negative effect on the adoption of health behaviors, and the effect became weaker with the addition of control variables. This suggests that the relationship between socioeconomic status and health behaviors is more complex in the group of youth entering the workforce.

Among other variables, some factors have a stabilizing effect on health behaviors. Gender is one such factor, and the positive regression coefficient for gender indicates that female youth are more health-conscious than male youth. The health status of those around them also influences the health behaviors of youth. It is common sense that the closer the youth group is socially to the subhealthy group, the more health behaviors are also valued. Perception of one's healthiness is also a factor, with youth believing that estimates of their healthiness negatively affect the health behaviors adopted. In other words, the healthier one feels, the less likely they are to adopt healthy behaviors. This suggests an important direction for future health promotion: emphasizing the need for healthy behaviors among youth who perceive themselves as healthier. Age had a limited effect on youth health behaviors. In the student population, age had no significant effect, suggesting that there was little variation in the importance placed on health behaviors among students of all ages. Among the non-student youth, the older the youth, the more they value health behaviors. In the full sample, age did not predict the importance that youth placed on health behaviors.

### 3.2 Reverse intergenerational influences on health behaviors

**Figure 1:**
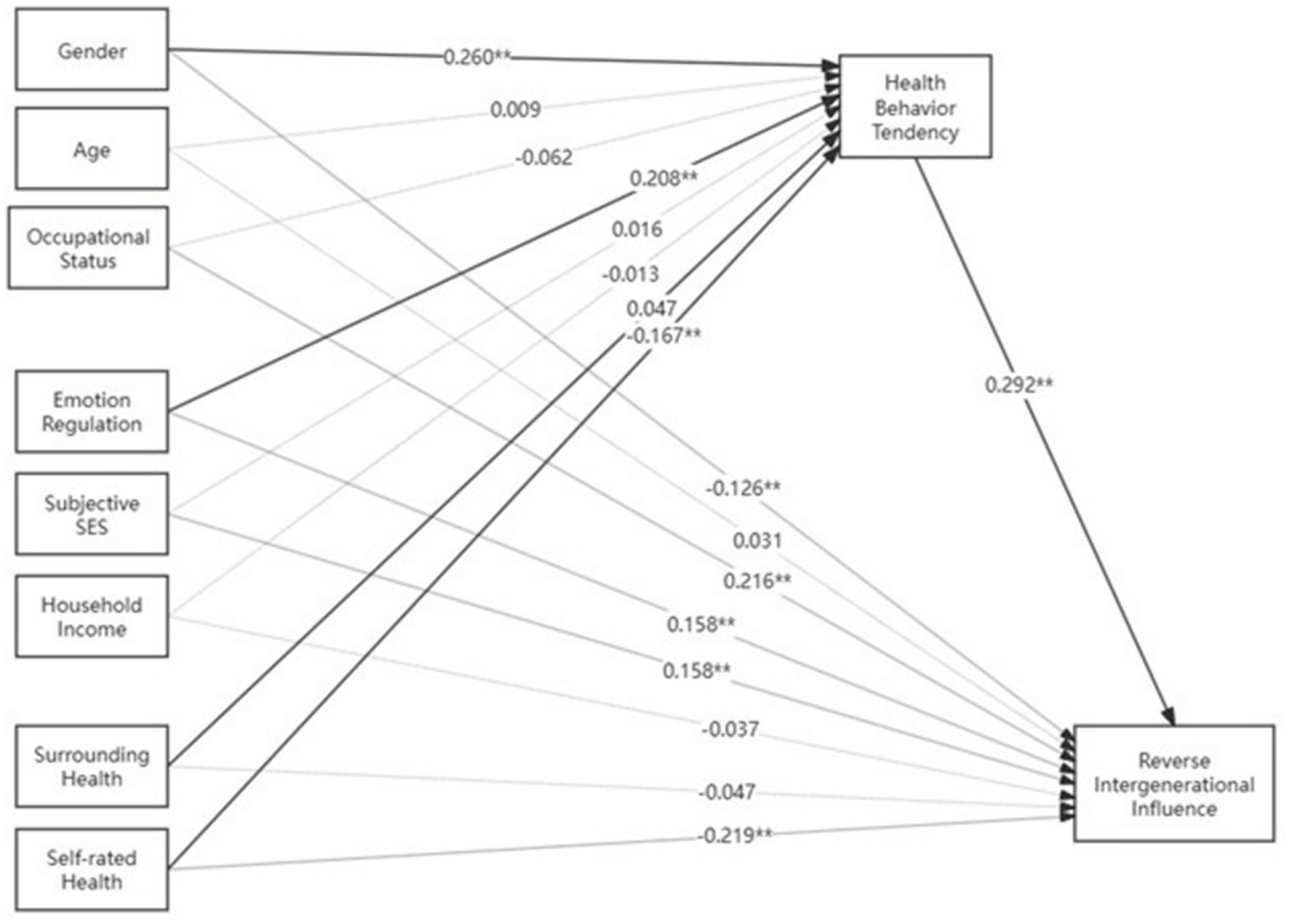
Mediation model examining the relationship between demographic factors, health-related variables, and reverse intergenerational influence mediated by health behavior tendency. *p < 0.05, **p < 0.01, ***p < 0.001. Standardized coefficients shown. Solid lines represent significant paths; dashed lines represent non-significant paths. See Table 5 for bootstrap mediation results.

Since previous analyses have demonstrated that emotional management ability, gender, surrounding people's health levels, and self-health level perception affect the generation of health behaviors. According to our prediction, the higher the youth health behavior tendency, that is, the more they value health behaviors, the more they will exert positive influence on elders' health behaviors. The model path diagram is shown in Figure 1, also see Table 5 for bootstrap results.

**Table 5:** Bootstrap mediation analysis results (*N* = 2,187, 5,000 replications).

**Variable**	**a-path^a^**	**b-path^b^**	**c'-path^c^**	**Indirect effect^d^**	**95% CI**	**Direct effect**	**95% CI**	**Total effect**	**Mediation type**
Emotional regulation	0.208^***^	0.292^***^	0.158^***^	0.061^***^	(0.043, 0.082)	0.158^***^	(0.116, 0.202)	0.219^***^	Partial
Gender	0.260^***^	0.292^***^	−0.126^**^	0.076^***^	(0.049, 0.109)	−0.126^**^	(−0.213, −0.039)	−0.050	Partial
Age	0.009	0.292^***^	0.031^***^	0.003	(−0.001, 0.006)	0.031^***^	(0.019, 0.043)	0.034^***^	No mediation
SES	0.016	0.292^***^	0.158^***^	0.005	(−0.002, 0.012)	0.158^***^	(0.127, 0.189)	0.163^***^	No mediation
Occupation	−0.062	0.292^***^	0.216^**^	−0.018	(−0.053, 0.013)	0.216^**^	(0.076, 0.351)	0.198^**^	No mediation
Surrounding health	0.079^***^	0.292^***^	0.047^*^	0.023^**^	(0.010, 0.040)	0.047^*^	(0.003, 0.091)	0.070^**^	Partial
Self-perceived health	−0.167^***^	0.292^***^	−0.219^***^	−0.049^***^	(−0.080, −0.027)	−0.219^***^	(−0.305, −0.134)	−0.268^***^	Partial
Household income	−0.013	0.292^***^	−0.037	−0.004	(−0.014, 0.006)	−0.037	(−0.075, 0.001)	−0.041^*^	No mediation

^a^a-path: Independent variable → Health behavior tendency.

^b^b-path: Health behavior tendency → Reverse intergenerational influence (constant across all variables).

^c^c'-path: Direct effect of independent variable → Reverse intergenerational influence.

^d^Indirect effect = a-path × b-path.

^*^p < 0.05.

^**^p < 0.01.

^***^p < 0.001.

CI = Confidence Interval based on bias-corrected bootstrap Indirect effects are significant when 95% CI does not include zero Partial mediation occurs when both direct and indirect effects are significant.

Research results show that in the above-described model, the effects of emotional management ability, gender, surrounding people's health levels, and perceived self-health levels on reverse intergenerational influence of health behaviors are mediated by youth health behavior tendencies. Among these, the direct effects of emotional management, self-health levels, and gender on reverse intergenerational influence of health behaviors are also significant, that is, youth health behavior tendency is partially mediating, indicating that these factors both influence reverse intergenerational transmission through youth health behavior tendencies and have independent direct influence pathways separate from health behaviors. The direct effect of surrounding people's health levels on reverse intergenerational influence is not significant and is completely mediated by health behavior perception. Additionally, subjective socioeconomic status and occupational factors have direct effects on reverse intergenerational influence. Specifically, the higher the subjective socioeconomic status, the stronger the reverse intergenerational influence of health behaviors; working groups have stronger reverse intergenerational influence on elders' health behaviors compared to student groups.

Among these, the mechanism of emotional management ability deserves attention. The direct effect of emotional management ability suggests that youth with strong emotional management abilities, even when their own health behavior tendencies are the same, can produce stronger reverse intergenerational influence effects when communicating with parents. Research suggests that adolescents with strong emotional management abilities can positively influence family relationships through effective communication (such as listening and emotional expression) (Denham, 2007), and this study's findings confirm similar mechanisms. The mediating effect of health behavior tendency suggests that good emotional regulation ability helps youth maintain health behaviors under stress, and their health behaviors have positive promoting effects on elders' health behaviors.

Additionally, subjective socioeconomic status and occupational status have direct effects on reverse intergenerational influence, suggesting that youth with better economic conditions often have more voice in families, and their suggestions are more likely to be valued and accepted by family members; similarly, compared to students, youth who are already working are considered more mature and reliable in families, therefore having stronger influence. Even within families, individuals evaluate the value of family members' behaviors and suggestions based on their social status and roles, not just based on specific behavioral performance, thereby affecting the social learning process (Bandura, 1977).

Bootstrap analysis with 5,000 replications confirmed the significance of key mediation pathways. The analysis revealed several significant indirect effects through youth health behavior tendency: Emotional regulation ability demonstrated a significant indirect effect [0.061, 95% CI (0.043, 0.082)], indicating that emotional regulation influences reverse intergenerational impact partially through health behavior tendency. Gender showed a significant indirect effect [0.076, 95% CI (0.049, 0.109)], demonstrating that gender differences in health behavior partially explain variations in intergenerational influence. Surrounding people's health had a significant indirect effect [0.023, 95% CI (0.010, 0.040)], confirming that environmental health cues influence intergenerational dynamics through individual health behaviors. Self-perceived health showed a significant negative indirect effect [−0.049, 95% CI [−0.080, −0.027]), indicating that better self-perceived health reduces intergenerational influence through lower health behavior adoption. Non-significant indirect effects were found for socioeconomic status [0.005, 95% CI (−0.002, 0.012)], age [0.003, 95% CI (−0.001, 0.006)], occupation [−0.018, 95% CI (−0.053, 0.013)], and income [−0.004, 95% CI (−0.014, 0.006)]. These results support the hypothesized mediation model, demonstrating that youth health behavior tendency serves as a critical mechanism linking individual and environmental factors to reverse intergenerational health influence. Four variables showed significant partial mediation effects, while three variables (SES, age, and occupation) influenced intergenerational outcomes only through direct pathways.

## 4 Discussion

### 4.1 Key factors influencing youth health behaviors

This study explored factors influencing youth health behaviors and their impact on reverse intergenerational influence using bootstrap mediation analysis. Bootstrap analysis with 5,000 replications confirmed four significant mediation pathways, providing robust statistical evidence for the proposed theoretical model of health “cultural feedback.” The findings demonstrate that youth health behavior tendency serves as a critical mediating mechanism linking individual characteristics and environmental factors to intergenerational health influence. Youth with stronger emotional management abilities demonstrated greater valuation of healthy behaviors, consistent with previous research confirming that unhealthy behaviors are closely associated with negative emotion accumulation (Xu et al., 2005). Given that young people's emotional management skills require improvement, they often exhibit heightened reactions to negative health information, adversely affecting their mental well-being (Research Group of the New Student Affairs Innovation Center, 2020). Our findings suggest that enhancing emotional management ability represents a feasible approach to addressing this issue.

Emotional regulation also demonstrates a direct positive effect on reverse intergenerational influence. Youth who effectively regulate emotions prove more successful in transmitting health knowledge to elders. Previous investigations have focused primarily on predetermined conditions such as family characteristics and personal resources (Wu, 2022), with limited attention to process-oriented factors, including variables affecting social information exchange efficiency, such as emotions and persuasive pathways (Pham and Avnet, 2004). Our study reveals that while emotional expression can impede cultural transmission from offspring to parents, proficient emotional management skills stabilize cognitive processes and promote youth initiative in health awareness transmission.

Additional influences on youth health behaviors included gender differences, with females demonstrating greater health behavior valuation than males, consistent with research indicating females' higher propensity for self-preservation behaviors (Yu, 2004). Youth surrounded by individuals in poor health, as well as those perceiving themselves as having poor health status, demonstrated increased attention to health behaviors, reflecting disease risk assessment processes (Xie et al., 2003).

Notably, socioeconomic status, occupation, and age factors exerted limited influence on youth health behavior adoption, suggesting that material inadequacy or social experience deficits do not impair health issue understanding among contemporary youth populations (Zaleskiewicz et al., 2013). However, while subjective socioeconomic status and employment status did not affect health behavior importance, they demonstrated direct effects on reverse intergenerational influence, consistent with research showing that offspring from affluent families or those employed possess greater family voice (Ekström, 2007).

Results reveal significant differences in socioeconomic status influence between student and working groups. Among student groups, subjective social status positively influences health behaviors, consistent with existing research findings. However, among working groups, a negative correlation emerges between income and health behaviors, demonstrating that the simplistic assumption of “more resources, better health” does not apply when explaining health behaviors. This negative association likely reflects competing time demands and occupational stress among higher income employed participants rather than measurement artifacts. Higher-income positions often involve greater work demands and time constraints that may impede health behavior maintenance, consistent with work-life conflict literature suggesting that career advancement can paradoxically reduce health-promoting activities (Burke and Greenglass, 2001). Work-related stress depletes individuals' self-regulation resources, thereby inhibiting health behaviors, indicating that the mechanism through which occupational status influences health behaviors may be more complex than previously understood.

Particularly noteworthy is our finding that individuals tend to adopt more health behaviors when they perceive worse health status among surrounding people. This result conforms to the risk cognition regulation mechanism of the health belief model rather than the “contagion” mechanism observed in social networks. When individuals observe people around them facing health threats, it appears to enhance subjective risk cognition regarding their own illness vulnerability, subsequently triggering positive health behaviors through strengthened defensive motivation. In mechanisms where social networks influence individual health behaviors and health status, values and norms typically play significant roles; however, the context of this study minimally involves such factors, which may explain why corresponding mechanisms cannot be demonstrated.

### 4.2 Mechanisms of health “cultural feedback”

The core contribution of this study lies in using empirical research to explore the specific mechanisms of reverse intergenerational influence of health behaviors, specifically studying the influencing factors of reverse intergenerational influence, and attempting to preliminarily establish a general theoretical model regarding health “cultural feedback.”

First, the key role of health behavior tendency as a mediating variable has been fully validated. The study found that variables such as emotional management ability, gender, surrounding people's health levels, and self-health perception all influence their guidance effectiveness on elders through the mediation of youth's own health behaviors. This mechanism conforms to the reciprocal determinism in social cognitive theory, where there is continuous interaction among individual cognition of health, personal behavioral habits, and external social environment, and these interactive mechanisms jointly shape health behavior patterns. In the pathway of youth influencing parents, health behavior tendency serves as a link connecting “individual traits” and “family influence.” This research result provides strong support for the applicability of “cultural feedback” theory in the health behavior field and further suggests that youth health behaviors and health concepts may be more important in shaping public health and social governance than previously thought.

Second, the research results reveal the direct and indirect effects of external variables on intergenerational influence. For example, although subjective socioeconomic status and occupational status do not significantly influence health behavior tendencies, they show significant positive effects on reverse intergenerational influence. This indicates that youth influence on family health behaviors is not only constrained by their health behaviors themselves but may also be closely related to their family roles and social identity. This finding supports “role strain theory,” namely that social role changes may bring corresponding changes in behavioral cognition and sense of responsibility. Particularly noteworthy is that emotional management ability is not only an important predictive factor for individual health behavior tendencies, but the mediating effect further suggests its role in intergenerational health behavior guidance. “Cultural feedback” is manifested not only in digital skill differences but also in “emotional pathways.” Individuals with higher emotional control abilities may more easily create good communication atmospheres in intergenerational interactions, thereby improving the acceptance of health advice.

Furthermore, this study found that the effect of surrounding people's health levels on reverse intergenerational influence is completely mediated by youth health behaviors, suggesting that social environmental cues do not directly drive youth to influence elders‘ health concepts and behaviors, but need to be transmitted through youth's own health behavior tendencies. This result conforms to the reciprocal determinism of social cognitive theory, namely that environmental factors (such as surrounding people's health levels) indirectly shape their effects on others' behaviors by influencing individual behavior tendencies. Specifically, when individuals observe poor health conditions of surrounding people, it may enhance their perception of health threats, thereby triggering their own health behaviors, and these behavioral tendencies subsequently indirectly influence elders‘ health concepts and practices through pathways such as information transmission, role model demonstration, or resource support. This mediating effect may suggest the importance of the processing process of social cues: the motivation to enhance health behaviors does not directly externalize into health guidance for elders but needs to be transformed into specific health practices to influence elders through intrafamily interactions. This may be because the health status of surrounding people, as external cues, needs to go through the internalization process of individuals' sense of responsibility for their own health and behavioral adjustment before extending to the intergenerational level.

In summary, the reverse intergenerational influence of youth health behaviors is subject to the joint action of individual-group multilevel factors, and these factors influence the influence on parents through health behavior tendency as a key mediating variable. This study constructs a mechanism model including “cognition-behavior-intrafamily structure,” providing specific descriptions of the complex associations among cognition, behavior, and intergenerational interaction in the “cultural feedback” mechanism.

### 4.3 Practical implications

The findings suggest practical pathways for health promotion that leverage cultural feedback mechanisms rather than traditional elder-focused interventions. Concrete interventions could include structured digital literacy training programs where young adults learn to identify health misinformation and develop communication strategies for family discussions. These programs would emphasize emotional regulation skills, given their demonstrated importance in both health behavior adoption and successful intergenerational influence. Mobile applications designed for intergenerational health sharing represent another avenue, enabling youth to curate verified health information while providing conversation starters that respect family dynamics. Community-based peer education programs could train young adults in evidence-based health communication, building on their established role as family “rumor busters” while providing structured approaches for addressing elder concerns about digital health information.

Implementation faces significant barriers rooted in traditional family hierarchies where knowledge typically flows from elders to youth. Successful interventions must navigate this resistance by framing young adults' contributions as information sharing with family rather than instruction, acknowledging elder experience while introducing contemporary health knowledge. The differential effects observed between working and student populations suggest that strategies require tailoring: working youth possess greater family influence but face time constraints, necessitating technology-mediated approaches, while students may need support in building confidence and perceived competence within family systems. Programs should also address the emotional dimensions of health communication, providing youth with skills to manage family conflicts when elders resist health advice and maintain long-term engagement without creating family tension. The investments in youth may yield multiplicative returns through intergenerational transmission, offering a more efficient approach to population health improvement in aging societies.

### 4.4 Limitations and future studies

This study has several limitations that warrant consideration. The cross-sectional design precludes definitive causal inferences regarding the relationships identified. While our findings demonstrate significant associations between emotional regulation, health behaviors, and reverse intergenerational influence, the temporal ordering of these relationships cannot be definitively established. However, the theoretical framework grounded in social cognitive theory provides strong conceptual support for the proposed directional relationships, particularly the pathway from individual characteristics through health behavior tendency to reverse intergenerational influence (Bandura, 1977). The mediation analyses further support these theoretically derived causal assumptions, though panel data studies would strengthen validation of temporal sequences.

Additionally, our reliance on subjective socioeconomic status measured through economic satisfaction may have influenced our findings in several ways. This single-item measure may not capture the multidimensional nature of SES, potentially underestimating the relationship between socioeconomic factors and health behaviors. Subjective SES can be influenced by reference group comparisons and individual aspirations beyond objective resources, which may explain the non-significant effects observed in our study. Future research should incorporate objective SES indicators including education level, occupation prestige, and household assets to provide a more comprehensive understanding of socioeconomic influences on health behaviors.

Future research should address these limitations through several approaches. Panel data studies with repeated observations would enable stronger causal inferences while remaining methodologically feasible. Multi-informant designs including both young adults and their parents would validate self-reported influence measures and provide comprehensive understanding of bidirectional family health dynamics. Although “cultural feedback” was first articulated within in China (Zhou, 1988), the within-family mechanisms identified in this study are unlikely to be culture-bound. Cross-cultural replication studies would establish the generalizability of cultural feedback mechanisms beyond the Chinese context.

## 5 Conclusion

This study examined determinants of young adults‘ health behaviors and their reverse intergenerational influence on older family members. Emotional regulation capacity emerged as the primary predictor of health behaviors, while socioeconomic factors showed limited direct effects. Young adults' health behaviors significantly predicted their influence on older generations through mechanisms including behavioral modeling, information transmission, and resource provision.

The findings validate the theoretical framework of health-related “cultural feedback,” demonstrating that young adults function as health promoters within family systems. Health behavior tendency serves as a critical mediator linking individual characteristics to intergenerational influence. These results suggest that interventions targeting emotional regulation skills among young adults may generate amplified health promotion effects through intrafamilial transmission.

## Data Availability

The raw data supporting the conclusions of this article will be made available by the authors, without undue reservation.
